# Prediction models for lymph node metastasis in cervical cancer based on preoperative heart rate variability

**DOI:** 10.3389/fnins.2024.1275487

**Published:** 2024-02-12

**Authors:** Weizheng Guan, Yuling Wang, Huan Zhao, Hui Lu, Sai Zhang, Jian Liu, Bo Shi

**Affiliations:** ^1^School of Medical Imaging, Bengbu Medical University, Bengbu, Anhui, China; ^2^Department of Gynecologic Oncology, The First Affiliated Hospital, Bengbu Medical University, Bengbu, Anhui, China

**Keywords:** heart rate variability, cervical cancer, machine learning, lymph node metastasis, autonomic nervous system

## Abstract

**Background:**

The occurrence of lymph node metastasis (LNM) is one of the critical factors in determining the staging, treatment and prognosis of cervical cancer (CC). Heart rate variability (HRV) is associated with LNM in patients with CC. The purpose of this study was to validate the feasibility of machine learning (ML) models constructed with preoperative HRV as a feature of CC patients in predicting CC LNM.

**Methods:**

A total of 292 patients with pathologically confirmed CC admitted to the Department of Gynecological Oncology of the First Affiliated Hospital of Bengbu Medical University from November 2020 to September 2023 were included in the study. The patient’ preoperative 5-min electrocardiogram data were collected, and HRV time-domain, frequency-domain and non-linear analyses were subsequently performed, and six ML models were constructed based on 32 parameters. Model performance was assessed using the area under the receiver operating characteristic curve (AUC), accuracy, sensitivity, and specificity.

**Results:**

Among the 6 ML models, the random forest (RF) model showed the best predictive performance, as specified by the following metrics on the test set: AUC (0.852), accuracy (0.744), sensitivity (0.783), and specificity (0.785).

**Conclusion:**

The RF model built with preoperative HRV parameters showed superior performance in CC LNM prediction, but multicenter studies with larger datasets are needed to validate our findings, and the physiopathological mechanisms between HRV and CC LNM need to be further explored.

## Introduction

Cervical cancer (CC) is a common gynecologic malignancy worldwide and one of the leading causes of cancer-related deaths in women (Sung et al., 2021). Clinical treatment is usually administered according to the disease stage of patients. For example, early CC patients are often treated with surgery, while patients with locally advanced CC need to be considered for combined radiotherapy and chemotherapy (Bhatla et al., 2021; Burmeister et al., 2022; Pecorino et al., 2022; Mereu et al., 2023). In 2018, the International Federation of Gynecology and Obstetrics (FIGO) included lymph node status in the CC staging criteria (Kido and Nakamoto, 2021). Since then, the occurrence of lymph node metastasis (LNM) has become an important factor in determining the staging and treatment modalities of CC (Hou et al., 2020). In addition, LNM has been proven to be an important risk factor for CC recurrence and patient death (Polterauer et al., 2010; Moreira et al., 2020). Therefore, it is of great significance to accurately assess whether LNM occurs in CC patients before treatment to make the best treatment decision and prognosis assessment.

Imaging methods such as magnetic resonance imaging (MRI), computed tomography (CT), and ultrasound are the current preferred choice for early non-invasive detection of CC lymph node status. However, the assessment of lymph node involvement by imaging methods relies mainly on lymph node size and morphological features. False-positive results may occur when there is a combination of inflammation, tuberculosis and hyperplastic lymph node lesions, and false-negative results may occur when there are small metastatic lymph nodes or micrometastatic foci. In recent years, with the development of artificial intelligence technology, radiomics approaches have emerged and been widely studied in the prediction of preoperative LNM status in patients with colorectal, bladder, breast, and biliary tract cancers (Huang et al., 2016; Wu et al., 2018; Mao et al., 2020). However, for the early prediction models of CC LNM by radiomics, the reproducibility of the radiomics features and the robustness of the model remain to be demonstrated due to the complexity of the lymph node images and the delineation relies on the subjective judgment of the diagnostician (Ji et al., 2019).

The autonomic nervous system (ANS) is an important component of the tumor microenvironment, which is involved in and modifies the cancer process (Manganaro et al., 2021). Evidence suggests that the ANS interacts with the development of inflammation, immunity, and metastasis in a variety of cancers (Bautista and Krishnan, 2020; Kamiya et al., 2021). The ANS, characterized by heart rate variability (HRV), has been extensively studied in cancer prognostic assessment, and preoperative HRV has been shown to be strongly associated with LNM status in a variety of malignant tumors. For example, Hu et al. (2018) and Simó et al. (2018) found that HRV decreased with tumor progression in patients with gastric cancer and correlated with LNM. Wang et al. (2021) found that in CC patients, HRV was significantly lower in the LNM group than in the no LNM group, and this association was independent of confounding factors such as age. If HRV can be used as a feature variable to build machine learning (ML) models to predict CC LNM, it would help to simplify the examination method for LNM.

The main objective of this study was to establish ML models to predict lymph node status based on preoperative short-term HRV features in CC patients, thereby providing new ideas for the preoperative prediction of LNM in CC patients.

## Materials and methods

### Subjects

The study was approved by the Medical Ethics Committee of Bengbu Medical University (Bengbu, Anhui, China) (2023-14). The experimental process was performed in strict accordance with the ethical standards set out in the 1964 Declaration of Helsinki and its amendments. All patients were informed of the detailed purpose, process, risks and adverse effects of the experiment and signed an informed consent form.

The study subjects were 427 CC patients admitted to the Department of Gynecological Oncology of the First Affiliated Hospital of Bengbu Medical University from November 2020 to September 2023. The inclusion criteria were as follows: (1) CC confirmed by pathohistological examination (squamous pathological types) and (2) new-onset patients without surgical treatment, radiotherapy and chemotherapy. The exclusion criteria were as follows: (1) carcinoma *in situ*; (2) incomplete pathological data; (3) poor quality of electrocardiographic signals; and (4) ectopic beats >5% of all beats.

### Data collection and heart rate variability analysis

The 5-min supine electrocardiogram data of CC patients were collected 1 day before surgery using a single-lead miniature electrocardiograph (version 2.8.0, Healink-R211B, Healink Ltd., Bengbu, China) with the sampling rate of the electrocardiograph set to 400 Hz and the bandwidth of the signal set to 0.6–40 Hz. The patient was asked to keep quiet and breathe steadily, and lead V6 was used.

The Pan-Tompkins algorithm was used to extract the electrocardiographic R-R interval (RRI) time series (Pan and Tompkins, 1985). Artifacts caused by extraction techniques, interference, and ectopic beats were automatically corrected using a time-varying threshold algorithm (Lipponen and Tarvainen, 2019). HRV analysis was then performed to obtain a total of 32 HRV parameters.

Heart rate indicators included mean heart rate (MeanHR), standard deviation of heart rate (SDHR), minimum heart rate (MinHR), and maximum heart rate (MaxHR).

Time-domain indicators included the standard deviation of all normal-to-normal intervals (SDNN), root mean square of successive interval differences (RMSSD), number of successive RR interval pairs that differed by more than 50 ms (NN50), NN50 divided by the total number of RR intervals (pNN50), triangular interpolation of normal-to-normal intervals (TINN), RR interval triangular index (RRTi, sampling interval 1/128 s), deceleration capacity (DC), acceleration capacity (AC).

Frequency-domain indicators included very low frequency (VLF, 0–0.04 Hz), low frequency (LF, 0.04–0.15 Hz), high frequency (HF, 0.15–0.4 Hz), total power (Total, 0–0.4 Hz), ratio between LF and HF (LF/HF), and electrocardiogram-derived respiration (RESP).

Prior to the frequency-domain analysis, the RR interval sequences were uniformly resampled using the 4-HZ cubic spline interpolation, the spectral values were estimated based on the fast Fourier transform (FFT) method, and the power spectral densities of the RR interval time series were estimated using the FFT of the Welch periodogram method (150 s window width, 50% overlapping windows).

Non-linear indicators indicated approximate entropy (ApEn) (embedding dimension *m* = 2 and the tolerance value *r* = 0.2 SDNN); detrended fluctuation analysis, which was calculated at 4 ≤ *n* ≤ 12 (short-term fluctuation, α1), 13 ≤ *n* ≤ 64 (long-term fluctuation, α2), and correlation dimension (CD), (embedding dimension *m* = 2); recurrence plot analysis, including mean diagonal line length (Lmean), maximal diagonal line length (Lmax), recurrence rate (REC), determinism (DET) and Shannon entropy (ShanEn), (embedding dimension *m* = 10, embedding lag τ = 1, and threshold distance *r* = √*m* SD, where SD is the standard deviation of the R-R time series); and multiscale entropy (MSE) produced by using sample entropy (SampEn) as a function of the scale factor, with scale parameter values τ = 1, 2, 3, 4, and 5 (SampEn_MSE1, SampEn_MSE2, SampEn_MSE3, SampEn_MSE4, SampEn_MSE5).

The above analysis was performed with Kubios HRV Premium software (version 4.0.2, Kubios Oy, Kuopio, Finland).^1^

### Machine learning modeling

Since the original dataset exists LNM (+) and LNM (–) category imbalance problem, the LNM (+) and LNM (–) were matched in the ratio of 0.8:1 by using the synthetic minority over-sampling technique (SMOTE) in order to enhance the model performance. The dataset was divided into training and test sets at a ratio of 7:3. Six ML models adaptive boosting (AdaBoost), Gaussian naive Bayes (GNB), logistic regression (LR), random forest (RF), support vector machine (SVM) and XGBoost, were built for CC LNM status classification. We used 10-fold cross-validation to evaluate the performance of the models on the training set. The optimal model was selected and the classification performance on the test set was further evaluated using the area under receiver operating characteristic curve (AUC), accuracy, sensitivity and specificity. ML models were constructed and validated using Python (Version 3.9) and R programming language (Version 3.6.3).

### Statistical analysis

Different representations were applied according to different types of data: mean ± standard deviation for normal continuous data, median (first quartile, third quartile) for non-normal continuous data, and count (percentage) for count data. The Shapiro-Wilk test was used to test the distribution normality of continuous variables. Independent samples *t*-tests and Mann-Whitney *U*-tests were performed to compare continuous variables between two groups. The chi-square test was used to compare count data between two groups. SPSS Statistics 26.0 (IBM Corp., Chicago, IL, United States of America) software was used for statistical analysis. *P* < 0.05 was defined as a significant difference.

## Results

### Patient characteristics

This study finally included 292 CC patients with an age and body mass index (BMI) of 54.0 ± 10.9 years and 24.8 ± 3.2 kg/m^2^, respectively. All patients were divided into LNM (–) and LNM (+) groups based on histopathologic findings. The LNM (–) group included 230 patients, and the LNM (+) group included 62 patients. After statistical analysis, we found no significant differences between the LNM (–) and LNM (+) groups in terms of age, BMI, hypertension, diabetes, tubal ligation, menopausal status, and adjuvant chemotherapy (*P* > 0.05). Table 1 describes the basic clinical characteristics of CC patients.

**TABLE 1:** Clinical and demographic data.

Characteristics	Total (*N* = 292)	LNM (–) (*N* = 230)	LNM (+) (*N* = 62)	*P*-value
Age (years)	54.0 ± 10.9	54.2 ± 11.0	53.3 ± 10.6	0.557
BMI (kg/m^2^)	24.8 ± 3.2	24.8 ± 3.3	24.7 ± 3.0	0.759
Hypertension				0.164
No	231 (79.1)	178 (77.4)	53 (85.5)	
Yes	61 (20.9)	52 (22.6)	9 (14.5)	
Diabetes				0.657
No	277 (94.9)	217 (94.3)	60 (96.8)	
Yes	15 (5.1)	13 (5.7)	2 (3.2)	
Tubal ligation				0.540
No	169 (57.9)	131 (57.0)	38 (61.3)	
Yes	123 (42.1)	99 (43.0)	24 (38.7)	
Menopausal status				0.250
No	118 (40.4)	89 (38.7)	29 (46.8)	
Yes	174 (59.6)	141 (61.3)	33 (53.2)	
Adjuvant chemotherapy				0.787
No	261 (89.4)	205 (89.1)	56 (90.3)	
Yes	31 (10.6)	25 (10.9)	6 (9.7)	

Values are expressed as the mean ± standard deviation, or the number of patients (percentages). LNM, lymph node metastasis; N, number of individuals; BMI, body mass index.

Table 2 shows the statistical results of the HRV parameters in CC patients. No HRV parameter was significantly different (*P* > 0.05) between the CC LNM (–) and LNM (+) groups; these results were obtained from the analysis of the raw data of 292 CC patients.

**TABLE 2:** Differences in HRV indicators between the LNM (–) and LNM (+) groups.

HRV	LNM (–) (*N* = 230)	LNM (+) (*N* = 62)	*P*-value
MeanHR (bpm)	70.6 (64.5, 77.3)	70.9 (64.9, 76.1)	0.851
SDHR (bpm)	2.2 (1.8, 2.8)	2.2 (1.7, 2.8)	0.467
MinHR (bpm)	66.1 (60.6, 72.0)	66.3 (60.7, 71.2)	0.932
MaxHR (bpm)	76.6 (70.1, 83.5)	76.5 (69.4, 83.3)	0.922
SDNN (ms)	26.9 (20.9, 33.8)	24.7 (19.4, 34.6)	0.419
RMSSD (ms)	16.2 (11.8, 24.8)	16.1 (10.9, 23.9)	0.632
NN50 (beats)	1 (0, 10)	1 (0, 9)	0.905
pNN50 (%)	0.27 (0.00, 2.78)	0.14 (0.00, 2.87)	0.923
RRTi	7.28 (5.90, 8.94)	7.05 (5.21, 9.09)	0.491
TINN (ms)	132 (99, 165)	122 (94, 173)	0.522
DC (ms)	13.2 (9.2, 20.3)	13.0 (8.2, 20.3)	0.721
AC (ms)	−14.2 (−20.6, −9.5)	−13.7 (−20.7, −8.7)	0.572
VLF (ms^2^)	315 (158, 563)	268 (164, 587)	0.873
LF (ms^2^)	104 (52, 188)	84 (43, 173)	0.307
HF (ms^2^)	94 (49, 215)	82 (42, 229)	0.512
Total (ms^2^)	572 (324, 954)	471 (276, 919)	0.592
LF/HF	1.092 (0.562, 1.989)	1.030 (0.474, 2.298)	0.849
RESP (Hz)	0.28 ± 0.05	0.29 ± 0.06	0.250
ApEn	1.134 (1.066, 1.176)	1.124 (1.046, 1.167)	0.427
α1	1.002 ± 0.277	1.031 ± 0.297	0.480
α2	1.041 ± 0.192	1.092 ± 0.152	0.057
CD	0.463 (0.219, 0.880)	0.416 (0.162, 0.885)	0.650
Lmean (beats)	12.90 (10.39, 17.78)	13.27 (10.79, 16.37)	0.509
Lmax (beats)	288 (164, 351)	302 (175, 355)	0.661
REC (%)	37.49 ± 10.48	37.68 ± 9.83	0.899
DET (%)	98.69 (97.45, 99.35)	98.79 (97.50, 99.42)	0.539
ShanEn	3.372 ± 0.396	3.416 ± 0.354	0.424
SampEn_MSE1	1.533 (1.355, 1.665)	1.532 (1.278, 1.731)	0.994
SampEn_MSE2	1.494 (1.311, 1.671)	1.476 (1.273, 1.602)	0.433
SampEn_MSE3	1.402 ± 0.282	1.358 ± 0.214	0.247
SampEn_MSE4	1.408 ± 0.299	1.401 ± 0.252	0.853
SampEn_MSE5	1.464 (1.253, 1.664)	1.371 (1.187, 1.581)	0.152

Values are expressed as median (first quartile, third quartile) or mean ± standard deviation. HRV, heart rate variability; LNM, lymph node metastasis; N, number of individuals.

### Diagnostic performance of the six machine learning models

**FIGURE 1:**
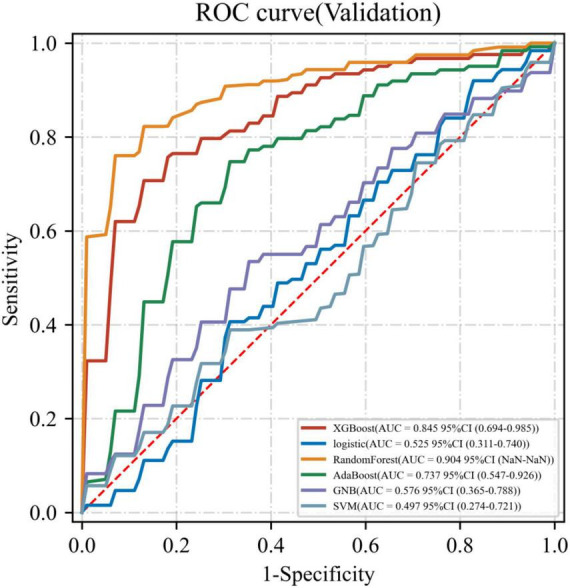
Receiver operating characteristic curve (ROC) for six ML models on the validation set.

**FIGURE 2:**
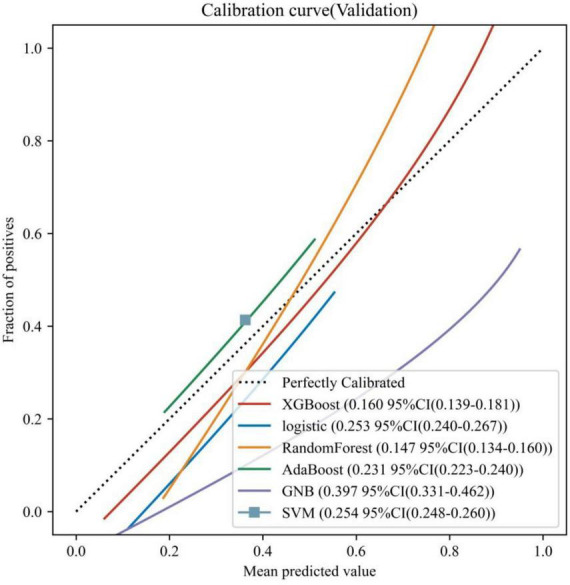
Calibration curves for the 6 ML models on the validation set. The dotted line represents the perfect calibration curve, i.e., the predicted probability matches the true probability perfectly. The numbers in the legend represent the Brier scores of the ML models; the smaller the Brier score, the closer the predicted probability of the ML model is to the true probability.

Six ML models were built based on the 32 HRV features, Figure 1 shows the AUC of 10-fold cross-validation for the 6 ML models on the validation set. Among them, the RF model had the highest AUC for 10-fold cross-validation (AUC = 0.904). The calibration curve (Figure 2) shows that among the six models, the RF model has the best fit between the predicted probability and the actual probability to discriminate LNM with a Brier score of 0.147.

**FIGURE 3:**
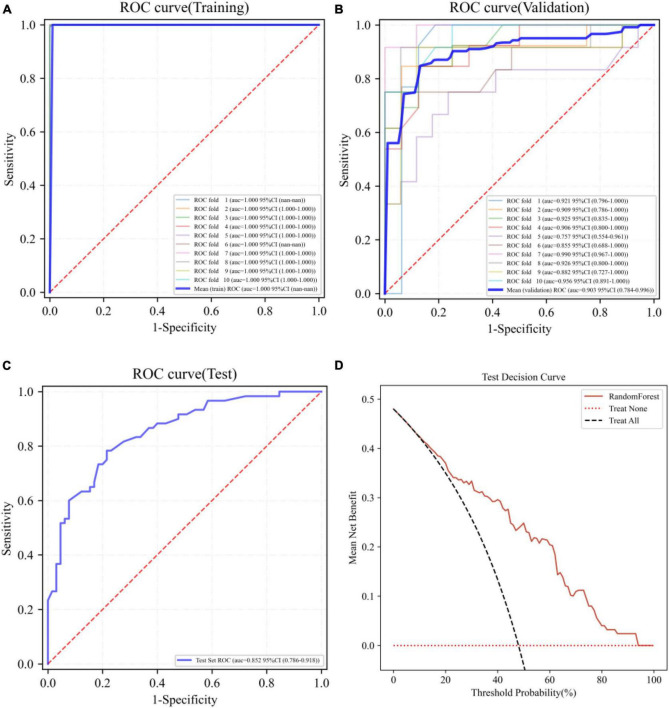
Receiver operating characteristic (ROC) curves of the RF model on the training set **(A)**, validation set **(B)**, test set **(C)**, and the decision curve on the test set **(D)**.

The best performing RF model was further tested using the test set. The ROC curves of the RF model in the training set, validation set, and test set are shown in Figures 3A–C, respectively. Figure 3D demonstrates the decision curve of the RF model in the test set, and the result shows that the RF model achieves a high net clinical benefit in most of the high risk threshold ranges. Table 3 shows the predictive metrics of the RF model on the training set, validation set, and test set.

**TABLE 3:** Predictive metrics of RF model on training set, validation set and test set.

	AUC	Accuracy	Sensitivity	Specificity
Training	1.000	0.996	1.000	0.995
Validation	0.903	0.844	0.854	0.909
Test	0.852	0.744	0.783	0.785

### Model interpretation

**FIGURE 4:**
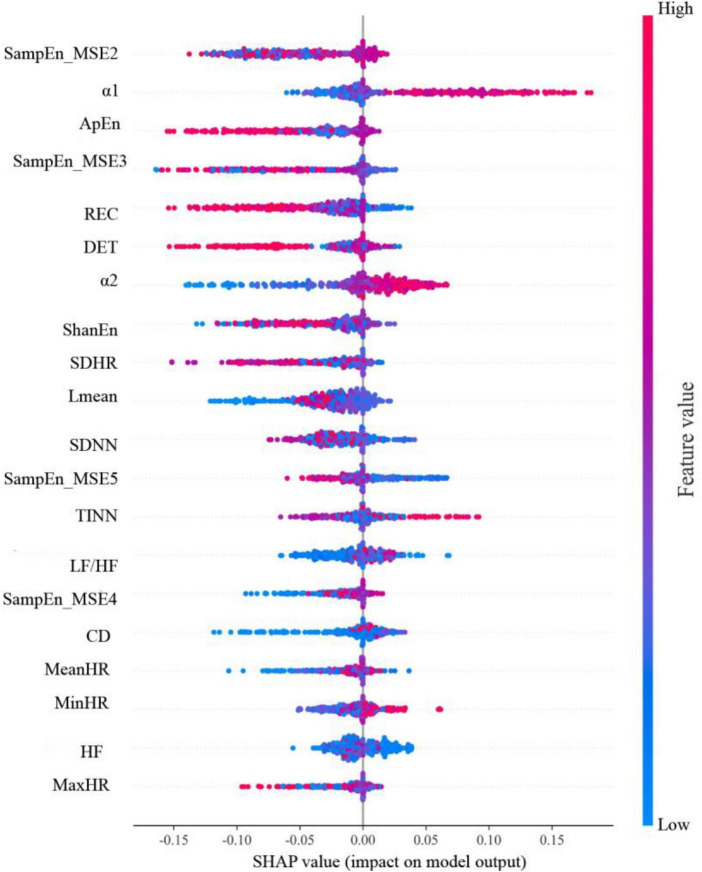
SHAP summary plot of 20 HRV parameters of the RF model.

The SHAP summary plot of the RF model is shown in Figure 4. The 20 features in the SHAP summary plot are arranged along the vertical axis in descending order of feature importance, with a higher position indicating a higher level of importance for the model to predict the LNM status. The features in order they are SampEn_MSE2, α1, ApEn, SampEn_MSE3, REC, DET, α2, ShanEn, SDHR, Lmean, SDNN, SampEn_MSE5, TINN, LF/HF, SampEn_MSE4, CD, MeanHR, MinHR, HF, MaxHR. For each feature, one point represents one patient. The horizontal axis is the SHAP value of the feature, the absolute value of which indicates the degree to which the feature affects the model output. Patients with higher SHAP values are at higher risk of developing LNM. Red indicates higher feature values, purple indicates feature values close to the overall mean, and blue indicates lower feature values.

## Discussion

In this study, we used HRV parameters obtained from the preoperative 5-min electrocardiogram data of CC patients to develop ML models for the classification of LNM status. Among them, the RF model performed the best with an AUC of 0.852, accuracy of 0.744, sensitivity of 0.783, and specificity of 0.785 on the test set. The results showed that the RF model based on preoperative HRV features could be used for CC LNM prediction.

In recent years, the use of ML techniques has been proposed to identify CC LNM, mainly using invasively obtained hematological parameters and/or non-invasive imaging parameters as model input features. For example, Ou et al. (2022) used pretreatment hematological parameters of CC patients to build an ML model to predict LNM in patients and built a Cforest model with a performance AUC of only 0.620. In addition, uncontrollable factors such as drugs and inflammation can affect the stability of hematological indicators, and different testing reagents and equipment can cause bias in the test results (Niu et al., 2020), all of which are not conducive to the popularization of this method. Arezzo et al. (2023) established LR and XGBoost models to predict LNM in patients with advanced CC using clinical data and pelvic MRI as the characteristic parameters, and the results showed that the XGBoost model demonstrated a better predictive performance (89% accuracy, 83% precision, 78% recall, and AUC 0.79). Although the model based on clinical features and MRI showed some performance improvement over the hematological parameter model, the method is more costly and the test is more time-consuming. In comparison, the ML model established with HRV parameters as features is much better than the above methods in prediction performance. In addition, HRV detection is a non-invasive method that is low cost, safe and easy to perform, which is also ideal for clinical promotion and application.

In ML-related studies, previous scholars have mostly used the *P*-value of statistical methods as a criterion for feature selection. However, this approach has certain pitfalls, i.e., the *P*-value is always manipulated to make a “one-size-fits-all” judgment with a threshold value of 0.05 or 0.01, which makes it easy to miss the potential contributions of features to the model prediction. In our study, although statistically significant differences in HRV metrics between the LNM (–) and LNM (+) groups were not observed, our test set of RF model based on HRV parameters reached an AUC of 0.852. The threshold for a significant difference (*P* < 0.05) is too strict and may ignore the contribution of some features to the classification (Guo et al., 2019). Traditional statistical methods may not be suitable for feature selection when modeling in ML, as ML methods can mine more potential relationships between data.

In this study, for the best predictive performance of the RF model, we used SHAP analysis to address the issue of model interpretability. The SHAP analysis showed that non-linear HRV parameters contributed more to the RF model. HRV analysis includes traditional time-domain, frequency-domain and non-linear analyses. Compared with traditional time-domain and frequency-domain parameters, non-linear parameters reflect the complexity of physiological signals better and can detect subtle changes in the early stages of disease (Busa and van Emmerik, 2016; Shi et al., 2017, 2019; Cui et al., 2020; Liao et al., 2022). The MSE complexity measure analysis method was proposed by Costa et al. (2002), which quantifies the non-linear dynamics of complex systems on multiple scales based on SampEn and measures signal complexity more comprehensively. Several studies have been conducted to apply the complexity indicators quantified by this method for disease prediction, classification and prognostic assessment (Lin et al., 2016; Frassineti et al., 2021; Tang et al., 2021; Yang et al., 2021; Liao et al., 2022). For example, Frassineti et al. (2021) found that MSE analysis had prescreening value in neonatal seizures. Tang et al. (2021) showed that MSE analysis was helpful for the identification of high-risk pulmonary hypertension patients. In contrast to other single time scale analyses, MSE can reflect an understanding of a range of time scales (Busa and van Emmerik, 2016). In practical applications, MSE analysis will be more accurate for long-duration electrocardiogram data; the length of the electrocardiogram data we analyzed was 5 min, so only 5 scales were analyzed. Interestingly, Zhang et al. (2021) used the 5 scales as well and noted that MSE showed greater discriminatory power in identifying coronary artery lesions. Combined with our findings, this implies that MSE may be promising indicators for detecting disease states, although the underlying mechanisms remain unclear. DFA provides an interpretation of shorter time series and can quantify the fractal behavior of complex dynamical systems (Peng et al., 1994; Nayak et al., 2018; Gu et al., 2022). In our results, we observed that the short-term fluctuation slope α1 in the DFA indicator played an important role in the RF model contribution, which may be related to the complex physiological mechanism behind it. The physiological context of DFA has been shown to be related to subtle interactions between sympathetic and vagal nerves (Tulppo et al., 2005; Beckers et al., 2006; Mandarano et al., 2022). As mentioned in the introduction, cancer progression involves dysfunction of ANS regulation, and LNM, as an important step in cancer progression, is associated with a combination of factors such as immune function, inflammatory response, and other factors, which can be influenced by the ANS through their regulation (Li et al., 2013; Le et al., 2016). In summary, the results of this study suggest that altered body complexity and ANS dysfunction are closely associated with CC LNM, but the specific mechanisms need to be further explored.

## Limitations

Our study also presents some limitations. First, there were some differences in the proportion of patients in the LNM (–) and LNM (+) groups in this study, and although we corrected for the sample imbalance using the SMOTE method, this may still have interfered with the results and affected the generalization ability of the model. Second, this study was a single-center study. Because HRV collection is prone to interference from the environment and other factors, external validation in a multicenter study is essential. Third, the physiopathological mechanisms between HRV parameters, especially non-linear parameters, and CC LNM need to be further explored.

## Conclusion

In conclusion, we investigated the feasibility of ML modeling using preoperative HRV parameters to predict CC LNM and demonstrated that the RF model may be a helpful detection tool. Being easy to implement, non-invasive and inexpensive, the technique is amenable to further clinical studies to refine our methodology and to determine the optimal application of the technique in clinical practice.

## Data availability statement

The raw data supporting the conclusions of this article will be made available by the authors, without undue reservation.

## Ethics statement

The studies involving humans were approved by the Medical Ethics Committee of Bengbu Medical University (Bengbu, Anhui, China) (2023-14). The participants provided their written informed consent to participate in this study.

## Author contributions

WG: Formal Analysis, Methodology, Writing – original draft. YW: Data curation, Methodology, Writing – review and editing. HZ: Formal Analysis, Methodology, Writing – review and editing. HL: Writing – original draft, Writing – review and editing. SZ: Writing – review and editing. JL: Conceptualization, Resources, Supervision, Writing – review and editing. BS: Conceptualization, Funding acquisition, Project administration, Resources, Writing – original draft, Writing – review and editing.

## Footnotes

1
https://www.kubios.com

## References

[B1] Arezzo F. Cormio G. Mongelli M. Cazzato G. Silvestris E. Kardhashi A. (2023). Machine learning applied to MRI evaluation for the detection of lymph node metastasis in patients with locally advanced cervical cancer treated with neoadjuvant chemotherapy. *Arch. Gynecol. Obstetr.* 307 1911–1919. 10.1007/s00404-022-06824-6 36370209

[B2] Bautista M. Krishnan A. (2020). The autonomic regulation of tumor growth and the missing links. *Front. Oncol.* 10:744. 10.3389/fonc.2020.00744 32477953 PMC7237572

[B3] Beckers F. Verheyden B. Aubert A. E. (2006). Aging and nonlinear heart rate control in a healthy population. *Am. J. Physiol.Heart Circ. Physiol.* 290 H2560–H2570. 10.1152/ajpheart.00903.2005 16373585

[B4] Bhatla N. Aoki D. Sharma D. N. Sankaranarayanan R. (2021). Cancer of the cervix uteri: 2021 update. *Int. J. Gynecol. Obstetr.* 155 (Suppl. 1), 28–44. 10.1002/ijgo.12611 34669203 PMC9298213

[B5] Burmeister C. A. Khan S. F. Schäfer G. Mbatani N. Adams T. Moodley J. (2022). Cervical cancer therapies: Current challenges and future perspectives. *Tumour Virus Res.* 13:200238. 10.1016/j.tvr.2022.200238 35460940 PMC9062473

[B6] Busa M. A. van Emmerik R. E. A. (2016). Multiscale entropy: A tool for understanding the complexity of postural control. *J. Sport Health Sci.* 5 44–51. 10.1016/j.jshs.2016.01.018 30356502 PMC6188573

[B7] Costa M. Goldberger A. L. Peng C. K. (2002). Multiscale entropy analysis of complex physiologic time series. *Phys. Rev. Lett.* 89:068102. 10.1103/PhysRevLett.89.068102 12190613

[B8] Cui X. Tian L. Li Z. Ren Z. Zha K. Wei X. (2020). On the variability of heart rate variability-evidence from prospective study of healthy young college students. *Entropy* 22:1302. 10.3390/e22111302 33263356 PMC7711844

[B9] Frassineti L. Lanatà A. Olmi B. Manfredi C. (2021). Multiscale entropy analysis of heart rate variability in neonatal patients with and without seizures. *Bioengineering* 8:122. 10.3390/bioengineering8090122 34562944 PMC8469929

[B10] Gu Z. Zarubin V. C. Mickley Steinmetz K. R. Martsberger C. (2022). Heart rate variability in healthy subjects during monitored. Short-term stress followed by 24-hour cardiac monitoring. *Front. Physiol.* 13:897284. 10.3389/fphys.2022.897284 35770191 PMC9234740

[B11] Guo H. Li Y. Mensah G. K. Xu Y. Chen J. Xiang J. (2019). Resting-state functional network scale effects and statistical significance-based feature selection in machine learning classification. *Comput. Math. Methods Med.* 2019:9108108. 10.1155/2019/9108108 31781290 PMC6875180

[B12] Hou L. Zhou W. Ren J. Du X. Xin L. Zhao X. (2020). Radiomics analysis of multiparametric MRI for the preoperative prediction of lymph node metastasis in cervical cancer. *Front. Oncol.* 10:1393. 10.3389/fonc.2020.01393 32974143 PMC7468409

[B13] Hu S. Lou J. Zhang Y. Chen P. (2018). Low heart rate variability relates to the progression of gastric cancer. *World J. Surg. Oncol.* 16:49. 10.1186/s12957-018-1348-z 29514707 PMC5842632

[B14] Huang Y. Q. Liang C. H. He L. Tian J. Liang C. S. Chen X. (2016). Development and validation of a Radiomics nomogram for preoperative prediction of lymph node metastasis in colorectal cancer. *J. Clin. Oncol.* 34 2157–2164. 27138577 10.1200/JCO.2015.65.9128

[B15] Ji G. W. Zhang Y. D. Zhang H. Zhu F. P. Wang K. Xia Y. X. (2019). Biliary tract cancer at CT: A radiomics-based model to predict lymph node metastasis and survival outcomes. *Radiology* 290 90–98. 10.1148/radiol.2018181408 30325283

[B16] Kamiya A. Hiyama T. Fujimura A. Yoshikawa S. (2021). Sympathetic and parasympathetic innervation in cancer: Therapeutic implications. *Clin. Aut. Res.* 31 165–178. 10.1007/s10286-020-00724-y 32926324

[B17] Kido A. Nakamoto Y. (2021). Implications of the new FIGO staging and the role of imaging in cervical cancer. *Br. J. Radiol.* 94:20201342. 10.1259/bjr.20201342 33989030 PMC9327757

[B18] Le C. P. Nowell C. J. Kim-Fuchs C. Botteri E. Hiller J. G. Ismail H. (2016). Chronic stress in mice remodels lymph vasculature to promote tumour cell dissemination. *Nat. Commun.* 7:10634. 10.1038/ncomms10634 26925549 PMC4773495

[B19] Li S. Sun Y. Gao D. (2013). Role of the nervous system in cancer metastasis. *Oncol. Lett.* 5 1101–1111. 10.3892/ol.2013.1168 23599747 PMC3629128

[B20] Liao T. E. Lo L. W. Lin Y. J. Chang S. L. Hu Y. F. Chung F. P. (2022). Nonlinear heart rate dynamics before and after paroxysmal atrial fibrillation events. *Acta Cardiol. Sin.* 38 594–600. 10.6515/ACS.202209_38(5).20220328A 36176370 PMC9479052

[B21] Lin Y. H. Lin C. Ho Y. H. Wu V. C. Lo M. T. Hung K. Y. (2016). Heart rhythm complexity impairment in patients undergoing peritoneal dialysis. *Sci. Rep.* 6:28202. 10.1038/srep28202 27324066 PMC4914979

[B22] Lipponen J. A. Tarvainen M. P. (2019). A robust algorithm for heart rate variability time series artefact correction using novel beat classification. *J. Med. Eng. Technol.* 43 173–181. 10.1080/03091902.2019.1640306 31314618

[B23] Mandarano P. Ossola P. Castiglioni P. Faini A. Marazzi P. Carsillo M. (2022). Heart rate fractality disruption as a footprint of subthreshold depressive symptoms in a healthy population. *Clin. Neuropsychiatry* 19 163–173. 10.36131/cnfioritieditore20220305 35821868 PMC9263681

[B24] Manganaro L. Nicolino G. M. Dolciami M. Martorana F. Stathis A. Colombo I. (2021). Radiomics in cervical and endometrial cancer. *Br. J. Radiol.* 94:20201314. 10.1259/bjr.20201314 34233456 PMC9327743

[B25] Mao N. Dai Y. Lin F. Ma H. Duan S. Xie H. (2020). Radiomics nomogram of DCE-MRI for the prediction of axillary lymph node metastasis in breast cancer. *Front. Oncol.* 10:541849. 10.3389/fonc.2020.541849 33381444 PMC7769044

[B26] Mereu L. Pecorino B. Ferrara M. Tomaselli V. Scibilia G. Scollo P. (2023). Neoadjuvant chemotherapy plus radical surgery in locally advanced cervical cancer: Retrospective single-center study. *Cancers* 15:5207. 10.3390/cancers15215207 37958381 PMC10648104

[B27] Moreira A. S. L. Cunha T. M. Esteves S. (2020). Cervical cancer recurrence – Can we predict the type of recurrence? *Diagn. Interv. Radiol.* 26 403–410. 10.5152/dir.2020.19437 32815522 PMC7490029

[B28] Nayak S. K. Bit A. Dey A. Mohapatra B. Pal K. (2018). A review on the nonlinear dynamical system analysis of electrocardiogram signal. *J. Healthc. Eng.* 2018:6920420. 10.1155/2018/6920420 29854361 PMC5954865

[B29] Niu L. Yao C. Wang Y. Sun Y. Xu J. Lin Y. (2020). Association between intermediate-acting neuromuscular-blocking agents and short-term postoperative outcomes in patients with gastric cancer. *Cancer Manag. Res.* 12 11391–11402. 10.2147/CMAR.S258016 33192096 PMC7654551

[B30] Ou Z. Mao W. Tan L. Yang Y. Liu S. Zhang Y. (2022). Prediction of postoperative pathologic risk factors in cervical cancer patients treated with radical hysterectomy by machine learning. *Curr. Oncol.* 29 9613–9629. 10.3390/curroncol29120755 36547169 PMC9776916

[B31] Pan J. Tompkins W. J. (1985). A real-time QRS detection algorithm. *IEEE Trans. Bio Med. Eng.* 32 230–236. 10.1109/TBME.1985.325532 3997178

[B32] Pecorino B. D’Agate M. G. Scibilia G. Scollo P. Giannini A. Di Donna M. C. (2022). Evaluation of surgical outcomes of abdominal radical hysterectomy and total laparoscopic radical hysterectomy for cervical cancer: A retrospective analysis of data collected before the LACC trial. *Int. J. Environ. Res. Public Health* 19:13176. 10.3390/ijerph192013176 36293758 PMC9603513

[B33] Peng C. K. Buldyrev S. V. Havlin S. Simons M. Stanley H. E. Goldberger A. L. (1994). Mosaic organization of DNA nucleotides. *Phys. Rev. E Stat. Phys. Plasmas Fluids Relat. Int. Top.* 49 1685–1689. 10.1103/physreve.49.1685 9961383

[B34] Polterauer S. Hefler L. Seebacher V. Rahhal J. Tempfer C. Horvat R. (2010). The impact of lymph node density on survival of cervical cancer patients. *Br. J. Cancer* 103 613–616. 10.1038/sj.bjc.6605801 20628380 PMC2938249

[B35] Shi B. Wang L. Yan C. Chen D. Liu M. Li P. (2019). Nonlinear heart rate variability biomarkers for gastric cancer severity: A pilot study. *Sci. Rep.* 9:13833. 10.1038/s41598-019-50358-y 31554856 PMC6761171

[B36] Shi B. Zhang Y. Yuan C. Wang S. Li P. (2017). Entropy analysis of short-term heartbeat interval time series during regular walking. *Entropy* 19:568. 10.3390/e19100568

[B37] Simó M. Navarro X. Yuste V. J. Bruna J. (2018). Autonomic nervous system and cancer. *Clin. Auton. Res.* 28 301–314. 10.1007/s10286-018-0523-1 29594605

[B38] Sung H. Ferlay J. Siegel R. L. Laversanne M. Soerjomataram I. Jemal A. (2021). Global cancer statistics 2020: GLOBOCAN estimates of incidence and mortality worldwide for 36 cancers in 185 countries. *CA A Cancer J. Clin.* 71 209–249. 10.3322/caac.21660 33538338

[B39] Tang S. Y. Ma H. P. Hung C. S. Kuo P. H. Lin C. Lo M. T. (2021). The value of heart rhythm complexity in identifying high-risk pulmonary hypertension patients. *Entropy* 23:753. 10.3390/e23060753 34203737 PMC8232109

[B40] Tulppo M. P. Kiviniemi A. M. Hautala A. J. Kallio M. Seppänen T. Mäkikallio T. H. (2005). Physiological background of the loss of fractal heart rate dynamics. *Circulation* 112 314–319. 10.1161/CIRCULATIONAHA.104.523712 16009791

[B41] Wang J. Liu J. Gao L. Li G. Sun Y. Shi B. (2021). Heart rate variability is an independent predictor of lymph node metastasis in patients with cervical cancer. *Cancer Manag. Res.* 13 8821–8830. 10.2147/CMAR.S336268 34853536 PMC8627856

[B42] Wu S. Zheng J. Li Y. Wu Z. Shi S. Huang M. (2018). Development and validation of an MRI-based Radiomics signature for the preoperative prediction of lymph node metastasis in bladder cancer. *EBiomedicine* 34 76–84. 10.1016/j.ebiom.2018.07.029 30078735 PMC6116473

[B43] Yang Z. Cheng T. Y. Deng J. Wang Z. Qin X. Fang X. (2021). Impairment of cardiac autonomic nerve function in pre-school children with intractable epilepsy. *Front. Neurol.* 12:632370. 10.3389/fneur.2021.632370 34248813 PMC8267887

[B44] Zhang C. K. Liu L. Wu W. J. Wang Y. Q. Yan H. X. Guo R. (2021). Identifying coronary artery lesions by feature analysis of radial pulse wave: A case-control study. *BioMed Res. Int.* 2021:5047501. 10.1155/2021/5047501 35005017 PMC8739924

